# Quality Characteristics, Anthocyanin Stability and Antioxidant Activity of Apple (*Malus domestica*) and Black Chokeberry (*Aronia melanocarpa*) Juice Blends

**DOI:** 10.3390/plants11152027

**Published:** 2022-08-03

**Authors:** Violeta Nour

**Affiliations:** Department of Horticulture & Food Science, University of Craiova, 13 AI Cuza Street, 200585 Craiova, Romania; vionor@yahoo.com; Tel.: +40-722-791-987

**Keywords:** apple (*Malus domestica*), aronia (*Aronia melanocarpa*), mixed juices, anthocyanins, polyphenolic compounds, antioxidant activity, heat treatment, storage, sensory evaluation

## Abstract

Black chokeberries are a valuable source of anthocyanins and other phenolic compounds, but they are underutilized due to their unpalatable astringent taste. The aim of this study was to determine the potential of using black chokeberry juice as a health-promoting ingredient in apple juice with a view to develop a new functional food product and to increase the dietary consumption of bioactive compounds. Mixed juices were prepared from apple (A) juice and black chokeberry (BC) juice at 95:5 (ABC5), 90:10 (ABC10), 85:15 (ABC15), and 80:20 (ABC20) volumetric ratios. Comparative studies on the effect of heat treatment (90 °C, 10 min) and storage (four months, 20 °C) on the physicochemical and antioxidant properties of apple, black chokeberry, and mixed juices were carried out. The soluble solids content, titratable acidity, total phenolic, total anthocyanin and ascorbic acid content, and antioxidant activity increased while the total soluble solids/titratable acidity ratio decreased with increasing addition levels of BC juice. Mixing A juice with BC juice at 95:5 and 90:10 volumetric ratios improved the color and enhanced the palatability and general acceptability of the juice. The percentage losses of anthocyanins and polyphenols registered after heat treatment and storage increased with increasing addition levels of BC juice.

## 1. Introduction

In recent years, the growing interest of consumers in maintaining healthy lifestyles has increased the efforts of researchers and manufacturers in terms of the development of functional foods. These are products that have been fortified or enriched to provide additional health-promoting benefits beyond the basic nutritional functions [1,2], able to enhance physiological function and to reduce the risk for diseases [3]. Epidemiological studies provide convincing evidence that regular or increased consumption of fruits and fruit products may promote general good health and well-being and may contribute to the decrease in the risk of various chronic diseases [4]. Fruit juices are among the most active functional food categories due to their freshness, sensory properties, and nutritional value. They are an excellent way to supply nutrients and bioactive compounds, including vitamins, minerals, fiber, polyphenols, carotenoids, chlorophylls, tannins, etc. [2,5]. At present, indigenous fruits, which are economically poorly explored, represent an interesting niche for the development of novel functional juices [5], able to meet customer demand for natural beverages, with new sensory attributes and health-promoting properties [6].

Apple juice is the second most popular juice worldwide, but apple ranks first in the European Union for juice processing [7,8] due to the large abundance of this fruit in the market and its long shelf-life [1]. Apple juice is commonly marketed a clear juice [9], but fresh cloudy apple juice is more popular among consumers for its softer taste, richer flavor, and higher nutritional value [10]. Cloudy apple juices are richer in dietary fiber, polyphenols, and mineral compounds and exhibit higher antioxidant activity than the clarified ones, as they are obtained without enzymatic and clarifying treatments [8]. However, cloudy apple juices are most susceptible to enzymatic browning through the oxidation of polyphenols catalyzed by polyphenol oxidase, which determines color instability during processing and storage [11,12].

Apples have a high nutraceutical value, being a source of nutrients and bioactive compounds such as ascorbic acid, polyphenols, and pectin. However, several comparative studies have reported that apples showed intermediate or even low values of phenolic content and antioxidant capacity as compared with other fruits [1]. Considering these and the wide availability, apple juice may be regarded as an ideal candidate for fortification or enrichment in order to increase its functionality.

Black chokeberry (*Aronia melanocarpa*, Rosaceae) fruit is one of the richest sources of phenolic compounds, especially anthocyanins, flavonols, flavanols, proanthocyanidins, and phenolic acids [13,14]. Native to North America, black chokeberry was introduced to Europe at the beginning of the twentieth century, but it has become popular only in the last decades, when the health-promoting benefits of its berries have been widely recognized based on the results of many preclinical and clinical studies proving their antioxidant, antimicrobial, antiviral, antidiabetic, antiatherosclerotic, hypotensive, antiplatelet, anti-inflammatory, and anti-cancer properties [15,16]. Although underutilized in the past, the constantly emerging evidence for their health-promoting properties has increased the popularity of black chokeberry fruits lately and has required their use in the production of juices, nectars, syrups, jams, fruit desserts, jellies, wines, and liqueurs or as a source of food-grade dye [14]. In recent years, black chokeberry has been widely cultivated in Europe, and the interest of food scientists and processors in its fruit has grown steadily related to the development of new functional foods [17]. Black chokeberry fruit shows the highest antioxidant activity among other berry and fruit species as a result of the high contents of ascorbic acid, anthocyanins, proanthocyanidins, and hydroxycinnamic acids [18]. The dark blue color of chokeberry fruit is a result of the high concentration of anthocyanins, primarily cyanidine 3-*O*-glucoside, 3-*O*-galactoside, 3-*O*-xyloside, and 3-*O*-arabinoside [14,16,19]. Black chokeberries are rarely consumed as fresh fruits or processed as individual juice because of their sour–bitter taste and astringent properties. As a result, black chokeberries are recommended to be added to other fruit products in order to increase their nutritional and functional properties or to improve their color [18].

The aim of this study was to determine the potential of using black chokeberry juice as a health-promoting ingredient in apple juice with a view to develop a new functional food product. Apple and black chokeberry juices were mixed in 95:5, 90:10, 85:15, and 80:20 volumetric ratios. The total soluble solids content, titratable acidity, pH, antioxidant activity, ascorbic acid, total anthocyanins, and total phenolic content were evaluated before and after heat processing and after 2- and 4-month storage periods. The acceptability of the juices was determined by sensory evaluation.

## 2. Results

After processing, apple juice had the lowest total soluble solids content (TSS) (13.57%) while the highest TSS value was recorded for black chokeberry juice (18.55%). Previous studies reported the content of soluble solids in the range of 18.15–25.61% in black chokeberry juices from different growing seasons [20] and of 13.30–20.99% [21] or 12.50–20.10% in commercial juices of different origin containing only black chokeberry [22]. The TSS values of the mixed juices measured immediately after processing ranged from 13.75% (ABC5) to 14.40% (ABC20) (Table 1).

TSS increased after heat treatment in all juices and continued to increase during four months storage, but the differences between the sampling times were not always significant at *p* < 0.05. However, in all juices, TSS was significantly (*p* < 0.05) higher after four months storage than at the time of processing. The higher TSS content after heat treatment and storage may be attributed to the hydrolysis of polysaccharides (starch and pectic substances) of juices into monosaccharides and other soluble solids under acidic conditions [23,24]. The results are in agreement with the findings of Khan et al. [25], who reported an increase in the TSS of mango–sea buckthorn blended juices after three months storage.

Titratable acidity (TA) and pH are important physicochemical parameters that influence the stability of bioactive compounds in fruit juices [26]. After processing, black chokeberry juice showed the highest titratable acidity (1.11% as malic acid) and apple juice the lowest (0.57% as malic acid) (Table 1). Tolić et al. [21] reported titratable acidity in the range of 0.89–1.06% as citric acid in chokeberry juices from different growing seasons. The higher the addition level of black chokeberry juice in the mix, the higher the titratable acidity of the blended juice. The TA values measured in blended juices immediately after processing ranged from 0.60% (ABC5) to 0.70% (ABC20) as malic acid. The titratable acidity decreased during storage in all juices while the heat treatment did not cause significant changes in titratable acidity. A decrease in the titratable acidity in juices during storage has been previously reported, and it has been attributed to the production of the acids in the acidic hydrolysis of polysaccharides [27,28,29].

Initially, the pH values of the mixed juices were in the range of 3.34–3.44. The pH slightly increased after heat treatment and then gradually decreased during four months storage in all juices. Similar decreases in pH values during storage have been reported by Khan et al. [25] in mango–sea buckthorn blended juices and have been attributed to the production of free acids as a result of the hydrolysis of the galacturonic acid methyl esters in pectin [30].

The TSS/TA ratio is a key characteristic of juice quality that impacts consumer acceptability and preferences [29]. Generally, a higher TSS/TA ratio is related to a higher sweeteness and more pleasant flavor of the juice and a higher acceptability among consumers [31]. Immediately after processing, the lowest TSS/TA ratio was found in the black chokeberry juice (15.81), indicating its lower suitability for consumption. The highest TSS/TA ratio was found in apple juice (23.81), but the addition of BC juice (TSS/TA = 15.81) determined the decline of this ratio. Similar results have been reported by Grobelna et al. [29], who found that the addition of blue honeysuckle berry juice (TSS/TA = 3.88) to apple juice (TSS/TA = 24.17) caused a decrease in the TSS/TA ratio. Lachowicz and Oszmiański [6] reported a decrease in the TSS/TA ratio of pear juice (TSS/TA = 21.21) as a result of the addition of cranberrybush juice (TSS/TA = 6.99).

The heat treatment determined the increase in the TSS/TA ratio as a result of the increase in TSS and the decrease in TA. In contrast, this ratio was reduced in each juice sample during storage due to increasing acidity. A decrease in the TTS/TA ratio was also reported by Khan et al. [25] for mango–sea buckthorn blended juice during storage.

Table 2 shows the total phenolic content (TPC) in A and BC juices and in mixed juice variants. Immediately after processing, TPC in A juice was 16.13 mg gallic acid equivalents (GAE)/100 mL, compared with 678.04 mg GAE/100 mL in BC juice. The addition of BC juice enriched the phenolic content of the apple juice by 3.09, 5.17, 7.22, and 8.83 times in ABC5, ABC10, ABC15, and ABC20, respectively.

Both heat treatment and storage significantly affected the phenolic content of all tested juices. Immediately after heat treatment, TPC decreased by 12.83%, 10.79%, 13.40%, 15.58%, 19.80%, and 23.70% in A, ABC5, ABC10, ABC15, ABC20, and BC juice, respectively. The higher the total phenolic content in the mixed juice, the higher the percentage of the phenolic loss due to heat treatment. TPC decreased during storage in all juices. After two months of storage, TPC decreased by 15.08% in A juice and by 15.17% in BC juice. The decrease in the TPC in the mixed juices during two months storage increased as the percentage of BC juice increased (9.02%, 12.58%, 13.25%, and 14.14% in ABC5, ABC10, ABC15, and ABC20, respectively). After four months storage TPC dropped even more; the greatest decrease in TPC was recorded in BC juice (34.93%) followed by ABC20 (29.89%) and was lowest in ABC5 (26.02%). A similar trend in TPC variation during storage was found by Grobelna et al. [29] in mixed juices made with the addition of blue honeysuckle berry juice to apple juice at 10, 20, and 30% levels and by Lachowicz and Oszmiański [6] in mixed juices obtained from pear juice after the addition of cranberrybush juice at 2.5, 5, 7.5, 10, and 25% levels.

Although ABC20 juice had the highest losses of phenolic compounds both in the heat treatment and during storage, at the end of four months storage, ABC20 juice had 2.44 times higher TPC (80.11 mg GAE/100 mL) compared to ABC5 juice. Note that, although twice as much BC juice was added to ABC20 as to ABC10, at the end of four months of storage, the content of phenolic compounds was only 1.5 times higher in ABC20 than in ABC10 (Table 2).

Black chokeberries are recognised as one of the richest sources of anthocyanins among other berry fruits, accounting between 25% [17] and 41% [32] of total polyphenols. They are represented mainly by 3-*O*-galactoside (68.9%), 3-*O*-glucoside (1.3%), 3-*O*-arabinoside (27.5%), and 3-*O*-xyloside (2.3%), while pelargonidin 3-*O*-galactoside and pelargonidin 3-*O*-arabinoside are detected only in trace amounts [33]. The total anthocyanin content of black chokeberry fruits can vary between 307 and 1480 mg/100 g FW [34]. Compared to black chokeberry fruits, apples have a low anthocyanin content, that, depending on the cultivar, may vary in the range of 1.3–12.3 mg/100 g [35]. In adition, anthocyanins are present in apples mainly in the skin; therefore, they are partially removed during juice processing.

Immediately after processing, a total anthocyanin content (TAC) of 3.04 mg of cyanidin 3-*O*-glucoside equivalents (CGE)/100 mL was found in apple juice while in chokeberry juice, TAC was on average 366.54 mg CGE/100 mL. In the mixed juices, TAC was essentially proportional to the black chokeberry juice concentration; the highest content of anthocyanins was recorded in ABC20 juice (75.78 mg CGE/100 mL) and the lowest in ABC5 juice (22.14 mg CGE/100 mL). Moreover, the addition of chokeberry juice leads to the red pigmentation of apple juice, making it more attractive to consumers.

TAC was significantly affected by the heat treatment in all juices. In the mixed juices, the highest decrease was found in ABC20 juice (24.09%) and the lowest in ABC5 juice (13.46%). The decrease in the anthocyanin content continued during storage. After four months storage, the highest decrease in TAC was recorded in BC juice (50.94%) followed by ABC20 juice (49.22%) and was the lowest in ABC5 juice (39.10%). Finally, after heat treatment and four months storage, only 52.71%, 47.74%, 42.64%, and 38.55% of the initial TAC remained in the ABC5, ABC10, ABC15, and ABC20 juices, respectively. The percentage losses of anthocyanins during storage were higher than those of total phenolics. Previous studies revealed that anthocyanins are very susceptible to degradation during processing and storage, and heat treatments affect the anthocyanins considerably more than the other phenols in terms of degradation [36]. Losses of total anthocyanins during storage have been attributed to polymerization, residual enzyme activity or condensation reactions of anthocyanins with other phenolics [37,38].

Fresh aronia berries are recognised as a good source of ascorbic acid, an essential vitamin in the human diet and a valuable natural antioxidant. Denev et al. [14] reported an ascorbic acid content (AAC) ranging between 37 and 92 mg/100 g FW (average 65.2 mg/100 g FW) in 23 aronia Bulgarian samples. Immediately after processing, BC juice contained 62.33 mg ascorbic acid/100 mL while A juice only 8.56 mg/100 mL. Adding BC juice to A juice increased the ascorbic acid content by 35.04% (ABC5), 66.12% (ABC10), 85.40% (ABC15), and 129.78% (ABC20) as compared with A juice. The ascorbic acid content registered high losses during heat treatment (between 48.01 and 53.3%), much higher than those recorded for the content of anthocyanins or phenolic compounds. After heat treatment and four months storage, only 16.46–17.39% of the initial AAC was found in the mixed juices.

The antioxidant activity (AA) of the tested juices was measured by the DPPH free radical-scavenging activity method, and the results are presented in Table 2. The antioxidant activity of chokeberry juice (19.77 mmol Trolox/100 mL) was 18.48 times higher than that of apple juice (1.07 mmol Trolox/100 mL). As a result, the addition of black chokeberry juice also determined a significant increase in antioxidant activity in the mixed juice. An increase in the free radical scavenging activity, of 1.97, 2.74, 3.71, and 4.63 times was observed in the ABC5, ABC10, ABC15, and ABC20 juices, respectively, after processing as compared with the A juice. Immediately after processing, the antioxidant activity was determined to be in the range of 1.78–3.66 mmol Trolox/100 mL in the mixed juices. The antioxidant activity decreased after heat treatment. As noted for TPC, the higher the addition level of BC juice, the higher the decrease in the DPPH antioxidant activity in the mixed juice (by 15.60, 20.33, 23.17, and 26.06% in ABC5, ABC10, ABC15, and ABC20 juices, respectively). As found for TPC, storage also contributed to the decrease in the antioxidant activity in all juices. After four months storage, AA measured by the DPPH method ranged from 1.12 in ABC5 to 2.11 mmol Trolox/100 mL in ABC20. The highest losses of DPPH antioxidant activity were recorded in the ABC20 juice (only 40.40% of the initial AA remained), whereas 52.86%, 47.51%, and 42.82% of initial AA remained in the ABC5, ABC10, and ABC15 juices, respectively. The results confirmed once again that the addition of berry juices with a high content of bioactive compounds and high antioxidant activity to popular fruit juices (e.g., apples or pears) even in small portions, leads to obtaining mixed juices with a high antioxidant content and potential health benefits.

The results of the color parameters of the juices immediately after processing, after heat treatment, and after two and four months storage at 20 °C are presented in Table 3. Immediately after processing, an average L* value of 83.99 was found for apple juice compared with only 33.81 for black chokeberry juice. In the blended juices, the L* values decreased as the percentage of added chokeberry juice increased. The heat treatment slightly decreased the L* value in apple juice, probably as a result of the enzymatic and nonenzymatic browning reactions, but the difference was not significant. Both heat treatment and four months storage determined a significant increase in the L* values in the blended juices with 10–20% added black chokeberry juice due to the decrease in the anthocyanin content, as previously shown. The blended juices with 10% and 15% added chokeberry juice (ABC10 and ABC15) showed the highest a* values (redness), higher than those determined in the mixtures with 20% black chokeberry juice addition (ABC20) or in chokeberry juice (BC). In addition, the blended juices with 5%, 10%, and 15% black chokberry juice had negative b* values, although the individual juices (both apple and black chokeberry juices) had positive b* values. The explanation for these findings may be that the color of the blended juice is determined not only by the pigment content of the individual juices from which they originate or by the mixing ratio but also by the interaction of pigments with each other or with other compounds and, last but not least, by the pH of the mixture. The environmental pH is decisive for the color shade and stability of anthocyanins, being well known, for example, the color changes of anthocyanins depending on the pH [39]. The a* values of the juices during both heat treatment and four months storage (Table 3) showed a general decreasing trend while b* values presented an increasing trend (decrease in redness and blueness), probably as a result of the anthocyanin losses resulting from the degradative processes.

**Figure 1:**
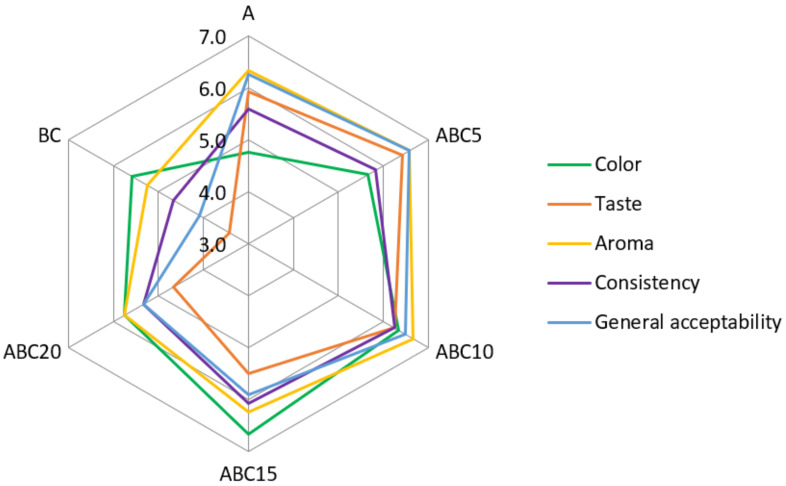
Sensory analysis of the apple, black chokeberry, and mixed juices. A—Apple juice; BC—black chokeberry juice; ABC5—mixed juice A:BC = 95:5 (*v*/*v*); ABC10—mixed juice A:BC = 90:10 (*v*/*v*); ABC15—mixed juice A:BC = 85:15 (*v*/*v*); ABC20—mixed juice A:BC = 80:20 (*v*/*v*).

The results of the sensory evaluation of singular and mixed juices from apple and black chokeberry are presented in Figure 1. The addition of aronia juice to apple juice determined statistically significant effects on sensory attributes such as color, taste, aroma, consistency, and general acceptability.

In terms of color, A juice received the lowest score (4.75) while ABC15 juice received the highest score (6.67). Due to the high content of anthocyanins, aronia juice has a dark purple color, which positively contributes to the color of the mixed juices. Panelists appreciated the dark red color of the mixed juices containing 10% and 15% chokeberry juice with higher scores (6.33 and 6.67, respectively) as compared to the dark purple color of the whole chokeberry juice (5.58).

For aroma, BC juice received the lowest score (5.25) and the highest scores were given to the ABC10 (6.67) and ABC5 (6.58) juices, which optimally combine the unique fruity flavor of the apple juice with the bitter almond smell of the aronia juice, given mainly by amygdalin, a cyanogenic glycoside isolated from the berries [17]. The cyanogenic compounds are important from a food safety perspective as they can release hydrogen cyanide when digested [40]. Previous studies reported an amygdalin content of 20 g/kg for aronia berries, 5.8 g/L for aronia juice [17], and between 0.001 and 0.08 g/L for apple juice [41].

The phenolic composition, acidity, and sugar content are the main factors that influence the sensory attributes of the juices [42]. Concerning taste, ABC5 juice achieved the highest score (6.42), which was probably due to the lowest acidity value and the highest TSS/TA ratio among the mixed juices. The taste scores decreased with increasing percentage of aronia juice (6.25, 5.50, and 4.67 for ABC10, ABC15, and ABC20, respectively) probably due to the highly astringent taste of the aronia fruits, which is known to have a negative impact on consumer preference [43]. The high levels of proanthocyanidins, compounds belonging to the tannin group, and non-volatile organic acids are considered to be primarily responsible for the pronounced bitterness, sourness, and astringency (drying/puckering sensation in the oral cavity) of the aronia juice [44,45,46]. Polysaccharides may reduce the perception of astringent taste as a consequence of adsorption of polyphenols on the surface of polysaccharides [47] and their interaction through hydrophobic interactions and hydrogen bonds [48]. The high polysaccharide content of the apple tissue, mainly pectins, may help reduce the astringency from aronia in the mixed juices, leading to juices that are more attractive to consumers.

With respect to the consistency, BC juice achieved the lowest score (4.67) due to its high viscosity determined by the high content of soluble solids and colloidal substances, mainly soluble pectins [49]. Mixing aronia juice with apple juice helped reduce the viscosity of the mixed juice; as a result, ABC10 received the highest score for consistency (6.25), followed by ABC15 (6.08).

In terms of general acceptability, the highest evaluation was obtained by ABC5 (6.58) and ABC10 (6.50) juices followed by ABC15 juice (5.92). Significantly less acceptable (*p* < 0.05) were the BC (4.08) and ABC20 (5.33) juices. Chrubasik et al. [50] suggested that pure aronia products are not particularly popular because of the astringent taste of the raw berries and their smell of bitter almonds. A higher percentage of black chokeberry juice added to apple juice negatively influenced the taste, aroma, and consistency of the mixed juice, making it less attractive and accepted by consumers.

## 3. Materials and Methods

### 3.1. Reagents and Standards

Methanol, Folin–Ciocalteu reagent, gallic acid, 2,2-diphenyl-1-picrylhydrazyl (DPPH), gallic acid, ascorbic acid, 6-hydroxy-2,5,7,8-tetramethylchroman-2-carboxylic acid (Trolox), and sodium carbonate were purchased from Sigma–Aldrich (Taufkirchen, Germany).

### 3.2. Raw Materials

Apples (*Malus domestica* cv. Jonathan) at the maturity stage were obtained from a local market in October 2021. The fruits were transferred to the laboratory and stored for 24 h at 4 °C before processing. Fruits of similar size without diseases, physiological defects or physical damages were selected as raw materials. They were washed with tap water and drained.

Black chokeberry (*Aronia melanocarpa*) fruits were supplied from local growers in the full maturity stage in September 2021. The berries were spherical in shape, with an average weight of 0.6 g. They were purplish-black in color and had a strongly astringent sour–sweet taste. The fruits were cleaned, drained, frozen, and then stored at −18 °C in polyethylene bags.

### 3.3. Juice Processing and Storage

Prior to juice extraction, the apples were sliced, while the black chokeberry fruits were thawed at 4 °C overnight. The fruits were separately juiced using a household juice extractor (Bosch MES3500, 700 W) and then filtered through 4-layered cheese cloth and poured into clear polyethylene terephthalate (PET) bottles. Apple juice (A) and black chokeberry fruit juice (BC) were mixed in the following volumetric ratios (A:BC): 95:5 (ABC5), 90:10 (ABC10), 85:15 (ABC15), and 80:20 (ABC20). The six juices (A, BC, ABC5, ABC10, ABC15, and ABC20) were poured into 200 mL transparent glass bottles, sealed, and sterilized at 90 °C for 10 min. After rapid cooling, the juices were stored at room temperature (20 ± 2 °C) in the absence of light. They were analyzed immediately after processing, after the heat treatment, and also after 2 and four months of storage at 20 °C. The experiment was repeated twice, and the analyses were performed using three replicates.

### 3.4. Physicochemical Parameters

The content of total soluble solids (TSS) of the juices was determined using a digital refractometer (Hanna Instruments, Woonsocket, RI, USA). The measurement was performed using three replicates for each sample, and the results were expressed as a percentage (%).

The pH values of the juices were recorded with a digital pH meter (Hanna HI255, Padua, Italy) calibrated using buffer solutions of pH 4 and pH 7. The juices were manually stirred to ensure homogeneity. The titratable acidity was determined by potentiometric titration with 0.1 N NaOH solution using the pH meter, i.e., Hanna HI255 (Hanna Instruments, Padua, Italy), until a pH of 8.1 was reached. The titratable acidity was expressed as a percentage of malic acid.

### 3.5. Total Phenolic Content

Phenolic compounds were extracted from juices using 80% (*v*/*v*) methanol. The total phenolic content was assessed by using the Folin–Ciocalteu method according to Singleton et al. [51]. An aliquot of juice extract (0.1 mL) was mixed with 5 mL of distilled water and 0.5 mL of Folin–Ciocalteu reagent. After 3 min, 1.5 mL of sodium carbonate solution (20% *w*/*v*) was added, and the mixture was made up to 10 mL with distilled water. The mixture was stirred and placed for 30 min at 40 °C in the dark, after which the absorbance was measured at 765 nm using a Varian Cary 50 UV spectrophotometer (Varian Co., Palo Alto, CA, USA). The results were expressed as milligrams of gallic acid equivalents per 100 mL of juice (mg GAE/100 mL) based on a standard curve of gallic acid (0.05–0.25 mg/mL).

### 3.6. Total Anthocyanin Content

Anthocyanins were extracted from fruit juices with 0.1% HCl (*v*/*v*). The quantification of total anthocyanins was performed using the pH differential spectrophotometric method described by Lee et al. [52]. The extract was diluted separately with buffers of pH 1.0 (0.025 M, potassium chloride) and pH 4.5 (0.4 M, sodium acetate), and the absorbance of each dilution was measured with a Varian Cary 50 UV spectrophotometer (Varian Co., Palo Alto, CA, USA) at 510 and 700 nm. The total anthocyanin content was calculated using the following formula:

Total anthocyanins (mg CGE/L) = (A × MW × DF × 1000)/(ε × l)(1)

where A = (A_510 nm_ − A_700 nm_)pH 1.0 − (A_510 nm_ − A_700 nm_)pH 4.5; MW (molecular weight) = 449.2 g/mol for cyanidin 3-*O*-glucoside; DF = dilution factor of the samples; ε (molar absorbtivity of cyanidin 3-*O*-glucoside) = 29,600 L/(mol·cm); l = pathlength in cm. The results were expressed as milligrams of cyanidin 3-*O*-glucoside equivalents per 100 mL of juice (mg CGE/100 mL).

### 3.7. Ascorbic Acid Content

The content of L-ascorbic acid in the tested juices was determined by the reversed-phase high-performance liquid chromatography method described by Nour et al. [53] using the Surveyor Thermo Electron system (Thermo Electron Corporation, San Jose, CA, USA). The juices were filtered through a 0.45 μm pore size filter and injected into a C18 column (Hypersil Gold aQ, 25 cm × 4.6 mm, 5 μm). Elution was performed under isocratic conditions at a flow rate of 0.7 mL/min using phosphate buffer solution (50 mM KH_2_PO_4_, pH = 2.8) as the mobile phase. The absorbance was recorded at 254 nm, and results were expressed as milligrams per 100 mL of juice based on a standard calibration curve of ascorbic acid (0–50 mg/L).

### 3.8. DPPH Free Radical-Scavenging Activity

The DPPH free radical-scavenging activity was determined in the juices based on the method described by Oliveira et al. [54]. Briefly, 3 mL of DPPH methanolic solution (0.004%) was added to 50 μL of juice extract. The mixture was shaken vigorously and kept in the dark for 30 min. After incubation, the absorbance of the mixed solution and of the blank of the DPPH solution without juice was read at 517 nm with a Varian Cary 50 UV–VIS spectrophotometer (Varian Co., Palo Alto, CA, USA). The inhibition of the DPPH radical by the sample was calculated according to the following formula:

DPPH scavenging activity (%) = [1 − absorbance of sample/absorbance of blank] × 100. (2)

Trolox (6-hydroxy-2,5,7,8-tetramethylchroman-2-carboxylic acid) was used as a standard reference, and results were expressed as mmol Trolox per 100 mL of juice.

### 3.9. Juice Color Parameters

The color of the juices was evaluated by measuring the L* (lightness), a* (redness/greenness), and b* (yellowness/blueness) values of the CIEL*a*b* system using a Thermo Scientific Evolution 600 UV/VIS spectrophotometer calibrated against a white standard. The total color difference (ΔE) was calculated following the formula:

ΔE = [(L* − L_0_*)^2^ + (a* − a_0_*)^2^ + (b* − b_0_*)^2^]^1/2^ (3)

where the subscript ‘0’ indicates the color of the juice immediately after processing.

### 3.10. Sensory Evaluation

The sensory properties of the juices were evaluated after processing using a 7-point hedonic scale, with 1 meaning “dislike extremely” and 7 meaning “like extremely”. Sensory attributes included color, taste, aroma, consistency, and general acceptability of the juices. The panel consisted of twelve members selected among staff and master students of the Department of Food Science, University of Craiova (Craiova, Romania). Coded juice samples were randomly served to the panelists at room temperature (20 °C) in transparent glasses, together with water to rinse their mouth between sample tasting.

### 3.11. Statistical Data Evaluation

Results were expressed as means ± standard deviations. The means were compared by using the least significant difference (LSD) test, and differences at *p* < 0.05 were considered to be significant. The statistical analysis was performed using Statgraphics Centurion XVI software (StatPoint Technologies, Warrenton, VA, USA).

## 4. Conclusions

The addition of black chokeberry juice is an excellent way to enrich apple juice with healthful anthocyanins and to include in the diet a fruit juice with outstanding health benefits but that is difficult to accept by consumers, mainly due to its high astringency. By increasing the addition level of chokeberry juice in the mixed juice, the total phenolic, total anthocyanin, and ascorbic acid contents and the DPPH radical scavenging activity increased, while the TSS/TA ratio of the mixed juices decreased. Both heat treatment and storage resulted in significant reductions in the contents of phenolic compounds and anthocyanins and the antioxidant activity; the higher the level of addition of chokeberry juice to the apple juice, the higher the percentage of the losses. The results of the sensory evaluation showed that mixing apple juice with black chokeberry juice at 95:5 and 90:10 volumetric ratios improved the color and enhanced the palatability and general acceptability of the juice, leading to attractive natural juice mixtures.

## Figures and Tables

**Table 1:** Physicochemical parameters of the tested juices immediately after processing, after the heat treatment, and after four months of storage *.

Sampling Time	Samples	TSS (%)	TA (% as Malic Acid)	pH	TSS/TA
Immediately after processing	A	13.57 ± 0.14 ^aA^	0.57 ± 0.02 ^cA^	3.28 ± 0.04 ^bA^	23.81
	ABC5	13.75 ± 0.10 ^aB^	0.60 ± 0.03 ^cAB^	3.34 ± 0.02 ^cAB^	22.92
	ABC10	14.02 ± 0.13 ^aC^	0.64 ± 0.03 ^cBC^	3.38 ± 0.03 ^bBC^	21.91
	ABC15	14.32 ± 0.12 ^aD^	0.67 ± 0.03 ^cC^	3.41 ± 0.02 ^bCD^	21.22
	ABC20	14.50 ± 0.06 ^aE^	0.70 ± 0.04 ^cC^	3.44 ± 0.03 ^bD^	20.57
	BC	18.55 ± 0.08 ^aF^	1.11 ± 0.06 ^cD^	3.63 ± 0.04 ^bE^	15.81
Immediately after heat treatment	A	13.67 ± 0.12 ^abA^	0.54 ± 0.03 ^bcA^	3.34 ± 0.04 ^bA^	25.31
	ABC5	13.87 ± 0.05 ^bB^	0.56 ± 0.02 ^bcA^	3.39 ± 0.02 ^cAB^	24.77
	ABC10	14.15 ± 0.14 ^bC^	0.61 ± 0.04 ^bcAB^	3.41 ± 0.03 ^bBC^	23.20
	ABC15	14.40 ± 0.17 ^abD^	0.64 ± 0.04 ^bcB^	3.46 ± 0.02 ^bCD^	22.37
	ABC20	14.65 ± 0.08 ^bE^	0.66 ± 0.03 ^bcB^	3.49 ± 0.03 ^bD^	22.05
	BC	18.68 ± 0.13 ^abF^	1.05 ± 0.07 ^bcC^	3.72 ± 0.04 ^cE^	16.84
After two months storage at 20 °C	A	13.72 ± 0.12 ^bcA^	0.51 ± 0.02 ^abA^	3.28 ± 0.03 ^bA^	23.66
	ABC5	13.95 ± 0.10 ^bcB^	0.52 ± 0.02 ^abA^	3.31 ± 0.03 ^bAB^	23.64
	ABC10	14.27 ± 0.05 ^bC^	0.56 ± 0.03 ^abAB^	3.32 ± 0.02 ^aABC^	22.30
	ABC15	14.53 ± 0.08 ^bcD^	0.60 ± 0.04 ^abBC^	3.35 ± 0.03 ^aBC^	21.22
	ABC20	14.72 ± 0.08 ^bE^	0.63 ± 0.04 ^abC^	3.37 ± 0.04 ^aC^	21.88
	BC	18.77 ± 0.14 ^bcF^	0.97 ± 0.06 ^abD^	3.58 ± 0.03 ^bD^	16.15
After four months storage at 20 °C	A	13.82 ± 0.08 ^cA^	0.48 ± 0.02 ^aA^	3.17 ± 0.02 ^aA^	22.29
	ABC5	14.05 ± 0.10 ^cB^	0.50 ± 0.03 ^aAB^	3.25 ± 0.03 ^aAB^	22.30
	ABC10	14.33 ± 0.15 ^bC^	0.53 ± 0.02 ^aAB^	3.32 ± 0.02 ^aABC^	21.38
	ABC15	14.62 ± 0.11 ^cD^	0.55 ± 0.03 ^aBC^	3.37 ± 0.03 ^aBC^	20.42
	ABC20	14.80 ± 0.09 ^bE^	0.59 ± 0.03 ^aC^	3.40 ± 0.03 ^aC^	19.60
	BC	18.83 ± 0.12 ^cF^	0.92 ± 0.05 ^aD^	3.50 ± 0.02 ^aD^	15.78

* Values for the same sampling time followed by different lowercase letters are significantly different based on the least significant difference (LSD) test (*p* < 0.05). Values for the same juice followed by different uppercase letters are significantly different based on the least significant difference (LSD) test (*p* < 0.05). A—Apple juice; BC—black chokeberry juice; ABC5—mixed juice A:BC = 95:5 (*v*/*v*); ABC10—mixed juice A:BC = 90:10 (*v*/*v*); ABC15—mixed juice A:BC = 85:15 (*v*/*v*); ABC20—mixed juice A:BC = 80:20 (*v*/*v*).

**Table 2:** Total anthocyanin, total phenolic, and ascorbic acid contents and the DPPH radical scavenging activity of the tested juices immediately after processing, after the heat treatment, and after four months of storage *.

Sampling Time	Samples	Total Anthocyanin Content (mg CGE/100 mL)	Total Phenolic Content (mg GAE/100 mL)	Ascorbic Acid Content (mg/100 mL)	DPPH Radical Scavenging Activity (mmol Trolox/100 mL)
Immediately after processing	A	3.04 ± 0.18 ^aD^	16.13 ± 0.73 ^aD^	8.56 ± 0.38 ^aD^	1.07 ± 0.06 ^aD^
	ABC5	22.14 ± 0.98 ^bD^	49.84 ± 2.43 ^bD^	11.56 ± 0.48 ^bD^	2.11 ± 0.09 ^bD^
	ABC10	41.73 ± 1.87 ^cD^	83.36 ± 3.98 ^cD^	14.22 ± 0.66 ^cD^	2.93 ± 0.14 ^cD^
	ABC15	58.74 ± 2.68 ^dD^	116.42 ± 5.67 ^dD^	15.87 ± 0.62 ^cD^	3.97 ± 0.18 ^dD^
	ABC20	75.98 ± 3.65 ^eD^	142.49 ± 6.66 ^eD^	19.67 ± 0.89 ^dD^	4.95 ± 0.23 ^eD^
	BC	366.54 ± 15.32 ^fD^	678.04 ± 30.42 ^fD^	62.33 ± 2.86 ^eD^	19.77 ± 0.78 ^fD^
Immediately after heat treatment	A	2.62 ± 0.12 ^aC^	14.06 ± 0.64 ^aC^	4.45 ± 0.18 ^aC^	0.88 ± 0.05 ^aC^
	ABC5	19.16 ± 0.84 ^bC^	44.46 ± 2.26 ^bC^	5.72 ± 0.24 ^bC^	1.78 ± 0.08 ^bC^
	ABC10	34.51 ± 1.66 ^cC^	72.19 ± 3.46 ^cC^	6.64 ± 0.28 ^cC^	2.33 ± 0.12 ^cC^
	ABC15	46.47 ± 2.08 ^dC^	98.28 ± 3.97 ^dC^	7.63 ± 0.34 ^dC^	3.05 ± 0.16 ^dC^
	ABC20	57.68 ± 2.68 ^eC^	114.27 ± 4.98 ^eC^	9.38 ± 0.40 ^eC^	3.66 ± 0.16 ^eC^
	BC	272.33 ± 12.61 ^fC^	517.34 ± 18.55 ^fC^	29.11 ± 0.98 ^fC^	14.32 ± 0.68 ^fC^
After two months storage at 20 °C	A	1.71 ± 0.09 ^aB^	11.94 ± 0.46 ^aB^	2.72 ± 0.16 ^aB^	0.61 ± 0.04 ^aB^
	ABC5	15.09 ± 0.54 ^bB^	40.45 ± 1.78 ^bB^	3.43 ± 0.22 ^abB^	1.42 ± 0.08 ^bB^
	ABC10	26.27 ± 1.18 ^cB^	63.11 ± 2.88 ^cB^	3.96 ± 0.26 ^bcB^	1.82 ± 0.07 ^cB^
	ABC15	34.38 ± 1.62 ^dB^	85.26 ± 3.65 ^dB^	4.69 ± 0.19 ^cdB^	2.29 ± 0.10 ^dB^
	ABC20	41.30 ± 1.87 ^eB^	98.11 ± 4.56 ^eB^	5.44 ± 0.36 ^dB^	2.89 ± 0.15 ^eB^
	BC	191.86 ± 8.78 ^fB^	438.86 ± 17.88 ^fB^	18.77 ± 0.88 ^eB^	10.32 ± 0.48 ^fB^
After four months storage at 20 °C	A	1.10 ± 0.06 ^aA^	10.37 ± 0.41 ^aA^	1.56 ± 0.04 ^aA^	0.40 ± 0.02 ^aA^
	ABC5	11.67 ± 0.43 ^bA^	32.89 ± 1.71 ^bA^	1.99 ± 0.06 ^bA^	1.12 ± 0.05 ^bA^
	ABC10	19.93 ± 0.81 ^cA^	53.28 ± 2.80 ^cA^	2.34 ± 0.05 ^cA^	1.39 ± 0.06 ^cA^
	ABC15	25.05 ± 1.09 ^dA^	70.64 ± 3.23 ^dA^	2.76 ± 0.05 ^dA^	1.70 ± 0.08 ^dA^
	ABC20	29.29 ± 1.46 ^eA^	80.11 ± 3.78 ^eA^	3.34 ± 0.14 ^eA^	2.11 ± 0.11 ^eA^
	BC	133.61 ± 6.88 ^fA^	336.61 ± 15.68 ^fA^	9.86 ± 0.28 ^fA^	7.24 ± 0.32 ^fA^

* Values for the same sampling time followed by different lowercase letters are significantly different based on the least significant difference (LSD) test (*p* < 0.05); Values for the same juice followed by different uppercase letters are significantly different based on the least significant difference (LSD) test (*p* < 0.05). A—Apple juice; BC—black chokeberry juice; ABC5—mixed juice A:BC = 95:5 (*v*/*v*); ABC10—mixed juice A:BC = 90:10 (*v*/*v*); ABC15—mixed juice A:BC = 85:15 (*v*/*v*); ABC20—mixed juice A:BC = 80:20 (*v*/*v*).

**Table 3:** Color parameters of the tested juices immediately after processing, after the heat treatment, and after four months of storage *.

Sample	Color Values	Immediately after Processing	After Heat Treatment	After 2 Months Storage at 20 °C	After 4 Months Storage at 20 °C
A	L*	83.99 ± 3.71 ^a^	81.36 ± 2.37 ^a^	81.51 ± 2.34 ^a^	82.57 ± 2.13 ^a^
	a*	1.18 ± 0.40 ^b^	−0.13 ± 0.08 ^a^	−0.22 ± 0.07 ^a^	−0.33 ± 0.02 ^a^
	b*	9.83 ± 0.45 ^d^	7.08 ± 0.67 ^a^	8.22 ± 0.47 ^b^	8.87 ± 0.32 ^c^
	ΔE	-	4.02	3.26	2.28
ABC5	L*	59.81 ± 0.53 ^b^	58.53 ± 1.35 ^a^	60.58 ± 0.98 ^b^	62.17 ± 0.84 ^c^
	a*	24.94 ± 0.36 ^d^	20.55 ± 0.88 ^c^	19.48 ± 0.71 ^b^	18.04 ± 0.82 ^a^
	b*	−4.26 ± 0.22 ^a^	−2.35 ± 0.12 ^b^	−0.77 ± 0.08 ^c^	0.80 ± 0.09 ^d^
	ΔE	-	4.95	6.52	8.87
ABC10	L*	44.43 ± 2.91 ^a^	49.83 ± 2.06 ^b^	52.20 ± 2.11 ^b^	55.01 ± 1.18 ^c^
	a*	27.30 ± 1.42 ^d^	24.26 ± 0.74 ^c^	21.61 ± 0.48 ^b^	18.29 ± 0.59 ^a^
	b*	−2.61 ± 0.51 ^a^	−2.72 ± 0.38 ^a^	−0.50 ± 0.06 ^b^	1.29 ± 0.16 ^c^
	ΔE	-	6.20	9.86	14.43
ABC15	L*	45.74 ± 2.54 ^a^	49.41 ± 2.22 ^b^	51.88 ± 2.16 ^b^	55.31 ± 1.22 ^c^
	a*	26.88 ± 1.13 ^d^	22.69 ± 1.14 ^c^	19.48 ± 0.49 ^b^	15.67 ± 0.35 ^a^
	b*	−2.27 ± 1.21 ^a^	−2.03 ± 0.21 ^a^	−0.03 ± 0.32 ^b^	3.42 ± 0.23 ^c^
	ΔE	-	5.58	9.88	15.81
ABC20	L*	39.60 ± 3.30 ^a^	46.46 ± 2.06 ^b^	48.85 ± 1.92 ^bc^	51.37 ± 1.64 ^c^
	a*	26.00 ± 0.59 ^d^	21.99 ± 1.20 ^c^	19.47 ± 1.34 ^b^	16.57 ± 0.87 ^a^
	b*	0.31 ± 0.05 ^b^	−1.43 ± 0.26 ^a^	0.90 ± 0.28 ^c^	3.75 ± 0.22 ^d^
	ΔE	-	8.14	11.33	15.47
BC	L*	33.81 ± 1.18 ^a^	37.68 ± 0.79 ^b^	38.96 ± 1.25 ^b^	40.54 ± 1.63 ^c^
	a*	20.97 ± 0.82 ^d^	16.30 ± 0.42 ^c^	14.03 ± 0.72 ^b^	12.29 ± 0.27 ^a^
	b*	1.80 ± 0.19 ^b^	1.14 ± 0.09 ^a^	2.65 ± 0.12 ^c^	4.38 ± 0.20 ^d^
	ΔE	-	6.10	8.69	11.28

* Values followed by different letters are significantly different based on the least significant difference (LSD) test (*p* < 0.05). A—Apple juice; BC—black chokeberry juice; ABC5—mixed juice A:BC = 95:5 (*v*/*v*); ABC10—mixed juice A:BC = 90:10 (*v*/*v*); ABC15—mixed juice A:BC = 85:15 (*v*/*v*); ABC20—mixed juice A:BC = 80:20 (*v*/*v*).

## Data Availability

Not applicable.
